# A case of mycotic common iliac aneurysm in a patient with ventriculoperitoneal shunt

**DOI:** 10.1016/j.jvscit.2025.101734

**Published:** 2025-01-15

**Authors:** Joseph DiBello, Justin Smith, Michael Sheehan, Daniel Lamb, Joseph McShannic

**Affiliations:** a Division of General Surgery, Department of Surgery, Summa Health, Akron, OH; b Division of Vascular Surgery, Department of Surgery, Summa Health, Akron, OH

**Keywords:** Mycotic aneurysm, Pseudoaneurysm, Retroperitoneal infection, VP shunt seeding, Extra-anatomic bypass

## Abstract

Aortoiliac mycotic aneurysm is an uncommon and deadly condition associated with significant perioperative morbidity. We present a case of common iliac mycotic pseudoaneurysm managed with debridement, vessel ligation, and extra-anatomic reconstruction owing to systemic illness. Notably, the patient had an implanted ventriculoperitoneal shunt for a history of cerebral aneurysm and resulting hydrocephalus. This report discusses the ultimately successful management of a large aortoiliac mycotic aneurysm. The patient did contend with bacterial meningitis postoperatively, likely owing to an ascending infection of the aforementioned shunt.

Mycotic aneurysm, referring to any infected aneurysm, is an uncommon and morbid condition first described in 1885 by Dr William Osler.^1^ The incidence among aortoiliac aneurysms ranges from 0.65% to 1.35%.^2^^,^^3^ Management generally involves extra-anatomic bypass, so as to avoid placing graft into an infected field. We review a case of aortoiliac mycotic aneurysm managed with open ligation and axillobifemoral bypass. Proper consent was obtained from the patient to publish this case report and imaging.

## Case report

**Fig 1:**
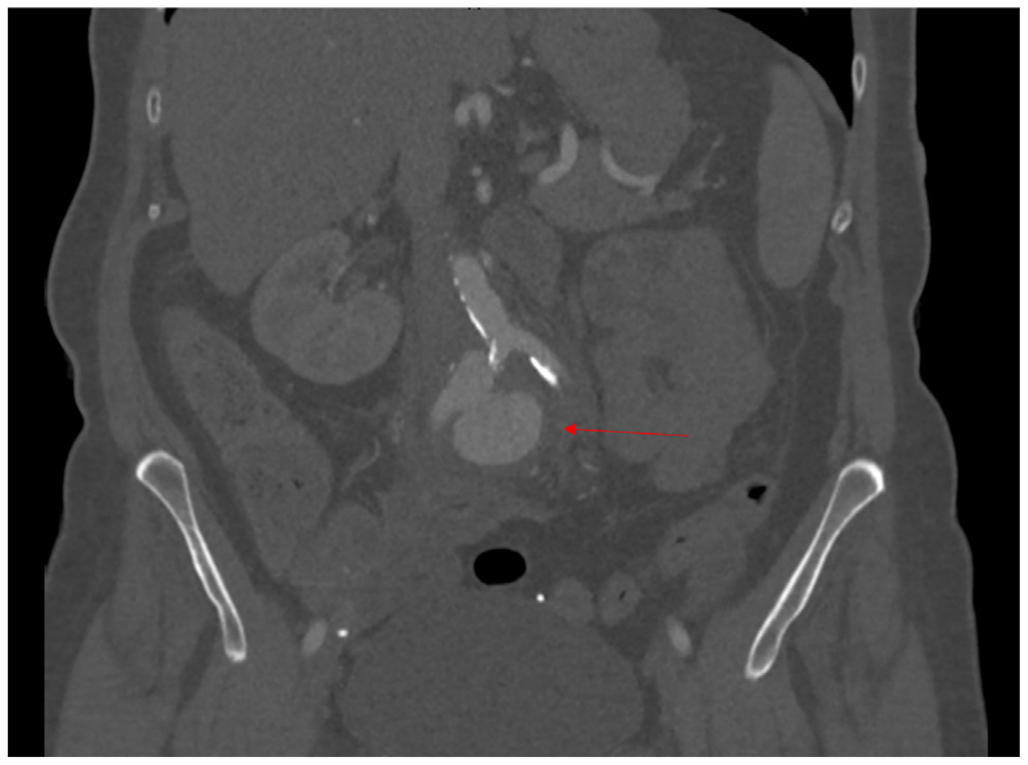
Computed tomography scan of right common iliac pseudoaneurysm (PSA) (red arrow).

**Fig 2:**
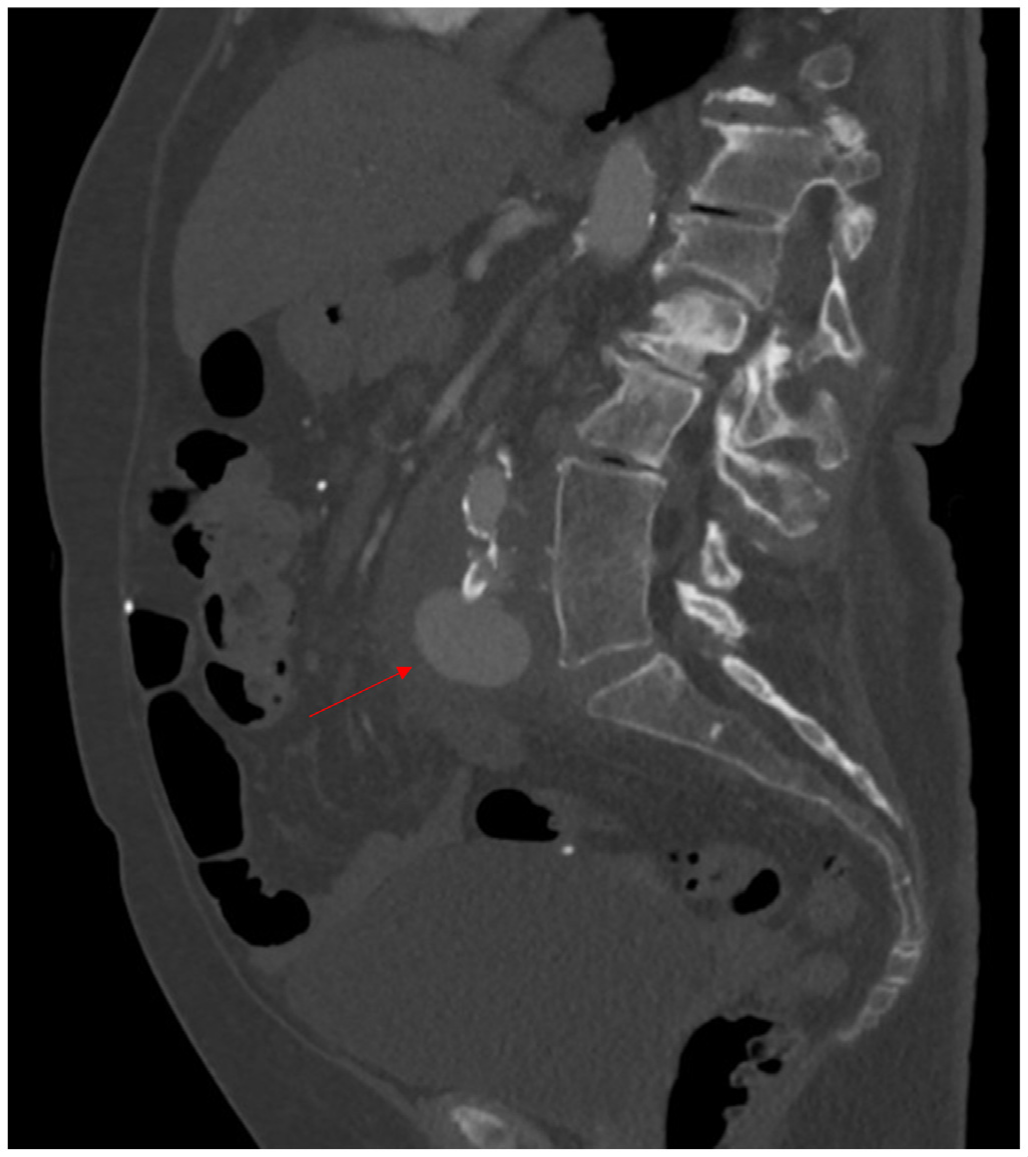
Computed tomography scan of right common iliac pseudoaneurysm (PSA), sagittal view (red arrow).

**Fig 3:**
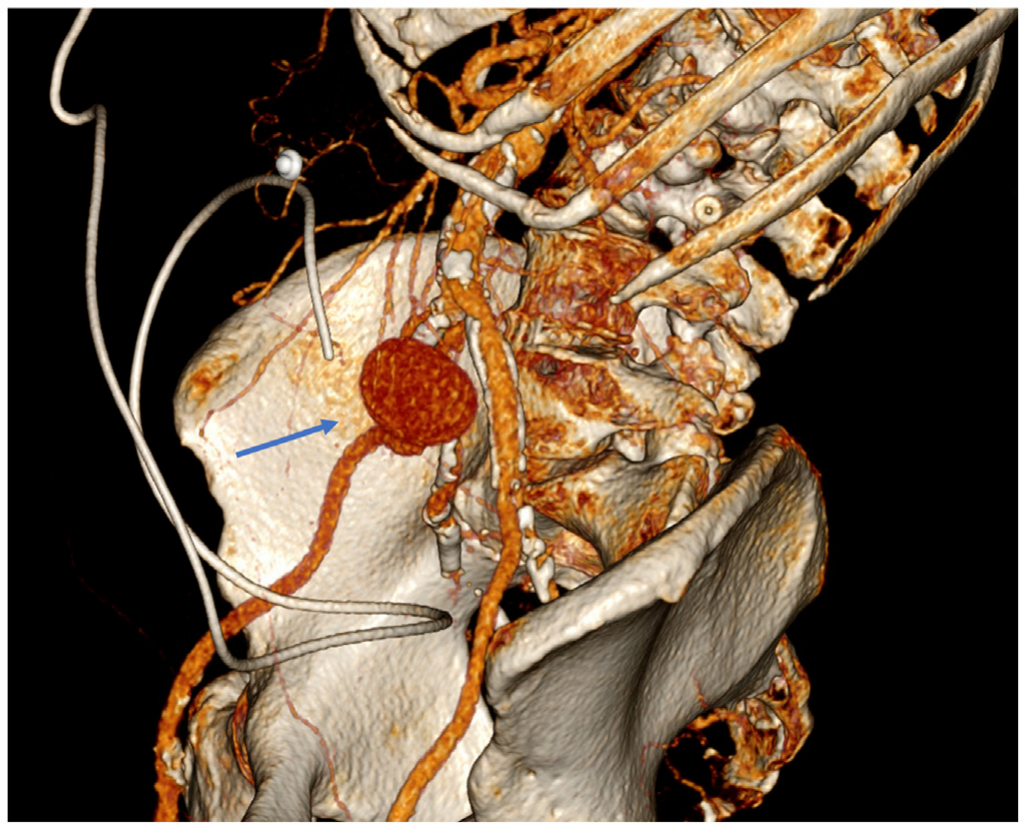
Three-dimensional rendering of the pseudoaneurysm (PSA) (blue arrow). The ventriculoperitoneal (VP) shunt is also shown.

The patient is a 76-year-old woman with a history of peripheral artery disease and ruptured cerebral aneurysm for which a ventriculoperitoneal (VP) shunt was placed 20 years prior. She initially presented with acute-onset low abdominal pain. Her computed tomography scan revealed periaortitis with retroperitoneal fibrosis, which was treated medically. However, she did not improve and was readmitted several weeks later with altered mental status and bacteremia. Repeat imaging showed worsening periaortic inflammation and the interval development of a 3.7-cm aneurysm originating from the proximal right common iliac artery (Fig 1, Fig 2, Fig 3). She was taken to the operating room by vascular surgery for urgent intervention and sepsis control. Given her extensive retroperitoneal inflammation, systemic signs of infection, and unavailability of a suitable cryopreserved conduit, we opted for extra-anatomic reconstruction. A right axillofemoral bypass was first constructed with a polytetrafluoroethylene graft. A right-to-left femoral-femoral bypass was then created, also using polytetrafluoroethylene. After closing these wounds, the abdomen was entered and the infrarenal abdominal aorta was exposed, revealing an aneurysm of the right common iliac system. After clamping, the large aneurysm was opened and debrided, revealing extensive purulence. The abdomen was irrigated thoroughly and the aorta and common iliac vessels were ligated with Prolene suture. A drain was left in the retroperitoneum. To mitigate contamination from the infected abdomen, the decision was made to exteriorize the VP shunt. The patient was admitted to the surgical intensive care unit postoperatively. Intraoperative cultures produced methicillin-sensitive *Staphylococcus aureus*, consistent with the preoperative blood cultures. The patient's postoperative course was complex. Although she initially progressed, subsequent setbacks included methicillin-sensitive *S aureus* bacterial meningitis, likely owing to shunt contamination. She required neurosurgical intervention and a temporary external ventricular drain until source control could be guaranteed. After extended-duration antimicrobial therapy and a prolonged surgical intensive care unit course, bacterial cultures cleared and the abdomen was felt to be sterile. A new VP shunt was created and placed in the upper abdomen laparoscopically.

Nearly 2 months after her index procedure, the patient was discharged to a rehabilitation hospital. At the 2-month follow-up, she was living semi-independently at home and progressing well without further complications. She was seen 18 months after discharge for management of her chronic lower extremity claudication symptoms, and continued to enjoy a good quality of life. Follow-up cross-sectional imaging revealed a patent axillobifemoral bypass and stable positioning of the VP shunt.

## Discussion

Mycotic aneurysms may arise from hematogenous spread, septic emboli, or, as in our case, the deterioration of the arterial wall by an abutting infection. An immunosuppressed state is a commonly referenced risk a factor, although it was not contributory in this case.^3^^,^^4^ The most common causative bacterium is *S aureus*; however, *Salmonella* species may be more prevalent in select regions of the world such as Asia.^5^^,^^6^ Diagnosis is made using laboratory tests, radiographic studies such as computed tomography angiography, and clinical presentation.^7^ Anti-microbial therapy and aggressive surgical debridement remain the key tenets of management.

Open surgical repair is generally used for the management of mycotic aortoiliac aneurysms, although endovascular repair has shown promise in select patients.^8^^,^^9^ After aneurysm resection and radical debridement of infected tissues, reconstruction can take place either in situ or with extra-anatomic bypass. Extra-anatomic reconstruction, often with an axillobifemoral graft, avoids the placement of graft in an infected field and has been the traditional approach used for a majority of patients.^10^, ^11^, ^12^ Drawbacks of extra-anatomic reconstruction include decreased long-term patency and the risk of aortic stump blowout.^13^ Alternatively, aortoiliac replacement can be performed in situ with a graft. In situ reconstruction is well-described and can be accomplished using a variety of conduits such as antibiotic-soaked prosthesis or cryopreserved allograft.^14^^,^^15^ Prospective studies comparing the in situ and extra-anatomic approaches are lacking; however, recent studies suggest that long-term outcomes after in situ reconstruction are acceptable.^16^^,^^17^

**Fig 4:**
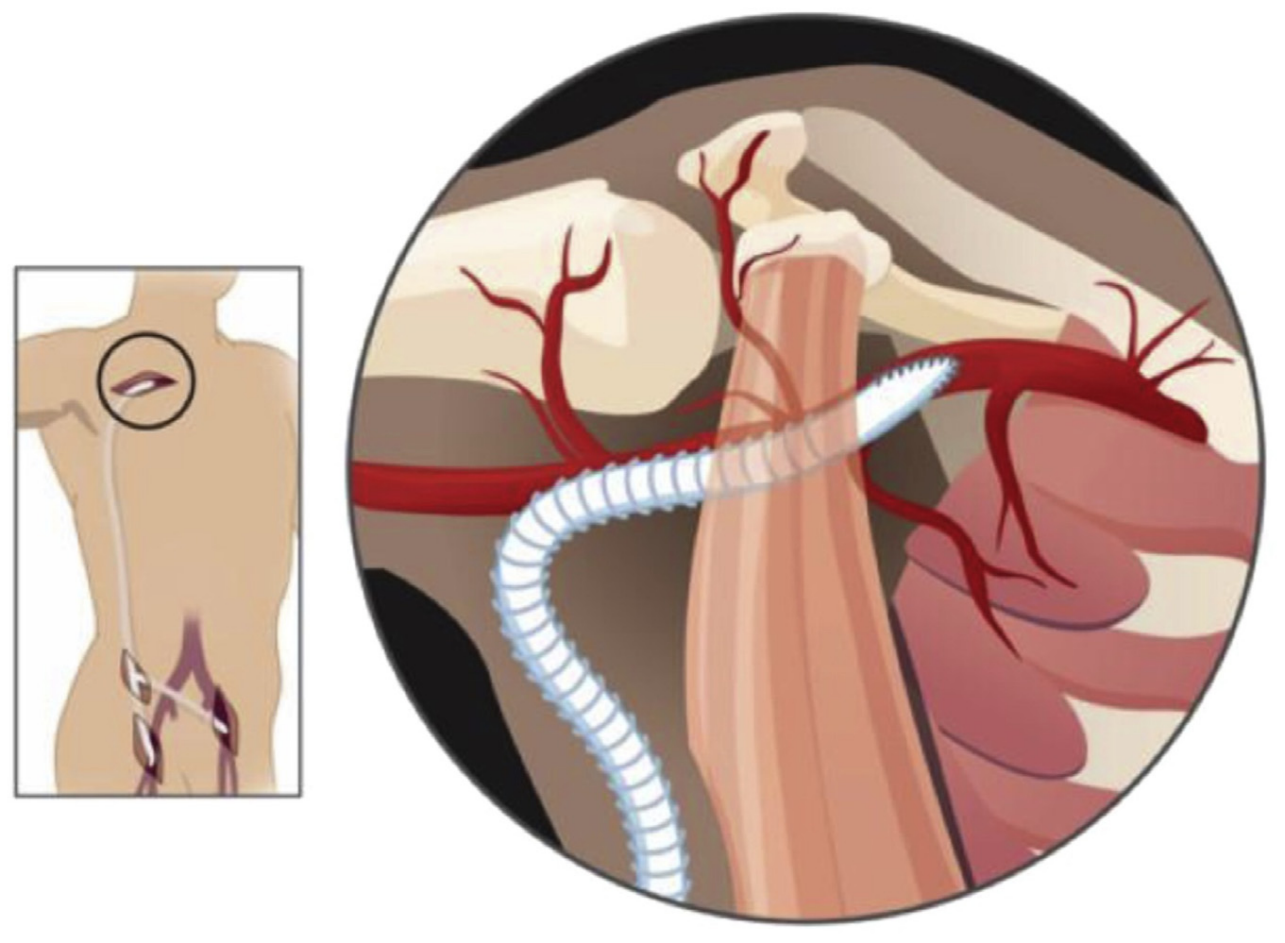
A rendering of an axillobifemoral bypass with emphasis on the proximal anastomosis. The arm is positioned at ninety degrees. A small amount of excess graft is left just distal to the anastomosis to allay any tension on the sutures from future hyperextension movements.

Reprinted from J Vasc Surg, 68(5), Samson RH, Showalter DP, Lepore MR Jr, Nair DG, Dorsay DA, Morales RE. Improved patency after axillofemoral bypass for aortoiliac occlusive disease, 1430-1437, Copyright (2018), with permission from Elsevier.

Nuances in surgical technique for axillobifemoral bypass may vary from surgeon to surgeon. Mitigating tension at the proximal anastomosis is a critical part of the procedure. Because it has been described before, we advise positioning the patient supine with the right arm abducted at 90°.^18^ This maneuver, along with leaving a measure of graft redundancy just distal to the anastomosis, will allow the patient to elevate their arm without placing tension on the suture line (Fig 4). Proximal graft dehiscence with arm hyperextension is well-described in the literature and is a complication we have managed in our practice.^19^^,^^20^

A 2023 case from *Clinical Practice and Cases in Emergency Medicine* reports a comparable instance of iliac mycotic pseudoaneurysm formation.^21^ This was thought to have developed in response to a pre-vertebral abscess after lumbar spine surgery. Interestingly, the pseudoaneurysm was managed endovascularly with a covered stent and an extended course of antibiotics.

The development of bacterial meningitis postoperatively was a major contributor to morbidity in this case. Because the intraoperative cultures matched the patient's subsequent cerebrospinal fluid cultures, we presume that seeding occurred from the abdomen via the VP shunt. It is also feasible that shunt compromise developed while the shunt remained exteriorized rather than as a direct result of the abdominal-based infection. The concept of seeding via VP shunt, whether infectious or malignant etiology, is rare but nonetheless described in the literature.^22^, ^23^, ^24^, ^25^

## Conclusions

Aortoiliac mycotic aneurysm is a life-threatening condition, with operative mortality as high as 20%.^26^ The cause of this patient's original retroperitoneal infection remains unclear, as there was no recent infectious history and no surgery since VP shunt placement 20 years prior. Nonetheless, the ongoing infection compromised the adjacent aortoiliac system, leading to pseudoaneurysm formation. Given the patient's significant purulence and systemic symptoms, we believe that open debridement with extra-anatomic reconstruction was her best option. Although retrograde seeding of the CSF via the VP shunt is uncommon, it likely contributed to the development of meningitis in this case. Additional prospective studies are needed to compare outcomes between open in situ, extra-anatomic bypass, and endovascular treatments of mycotic aneurysm.

## Funding

None.

## Disclosures

None.
